# Dunhuang Gancao Fuling Xingren decoction and its components alleviate CPT-11 induced intestinal mucositis by regulating gut microbiota related innate immunity and inflammatory response in *Drosophila* and mice

**DOI:** 10.1186/s13020-025-01279-8

**Published:** 2026-01-05

**Authors:** Jinhan Wu, Minghui Xiu, Xiaoqian Wang, Peihao Zhang, Yujie Qin, Jiangnan Li, Xiaolin Jiang, Yaoxing Duan, Yongqi Liu, Jianzheng He

**Affiliations:** 1 Gansu Province Research Center for Basic Disciplines of Dunhuang Medicine, Gansu University of Chinese Medicine, Lanzhou, 730000 China; 2 College of Public Health, Gansu University of Chinese Medicine, Lanzhou, 730000 China; 3 Key Laboratory of Dunhuang Medicine, Ministry of Education, Lanzhou, 730000 China; 4 Gansu Provincial Hospital, Lanzhou, 730000 China; 5 Research and Experimental Center, Gansu University of Chinese Medicine, Lanzhou, 730000 China

**Keywords:** Chemotherapy-induced intestinal mucositis, Dunhuang Gancao Fuling Xingren decoction, Molecular mechanism, Gut microbiota dysbiosis

## Abstract

**Background:**

Dunhuang Gancao Fuling Xingren decoction (GFXD) is a traditional formulation derived from the Dunhuang Ancient Medical Prescriptions, has been historically utilized for its immunomodulatory and anti-inflammatory properties. However, the protective effect against irinotecan (CPT-11)-induced intestinal mucositis (CIM) remains poorly elucidated.

**Purpose:**

To investigate the therapeutic efficacy of GFXD in alleviating CIM and elucidate its underlying mechanism and components using *Drosophila melanogaster* and *C57BL/6 J* mouse models.

**Methods:**

The therapeutic efficacy of GFXD was assessed in both *Drosophila* and mouse models by phenotype assay, hematoxylin and eosin (H&E) staining, and Alcian blue-periodic acid schiff (AB-PAS) staining. Transcriptomic profiling combined with 16S rRNA sequencing were employed to identify potential mechanisms of GFXD regulating CPT-11-induced mucositis. Cytokine levels were measured using ELISA, while the expression levels of key signaling pathways, including Toll-Imd and JAK-STAT pathways were analyzed via qRT-PCR, immunofluorescence, fecal microbiota transplantation (FMT) experiment, and antibiotic treatment. Furthermore, functional components of GFXD were characterized via liquid chromatography-mass spectrometry (LC–MS), and their efficacy was validated in CPT-11-treated *Drosophila*.

**Results:**

GFXD significantly mitigated CPT-11-induced systemic and intestinal damage in *Drosophila*, evidenced by improved survival rate, restored digestive function, elongated intestinal length, reduced acid–base imbalance, and enhanced epithelial and stem cell proliferation. In mice, GFXD alleviated mucositis symptoms, attenuated histopathological damage, and normalized inflammatory cytokine levels. Mechanistically, GFXD suppressed gut microbiota dysbiosis by enriching probiotics (*Lactobacillus, Prevotella*) and reducing pathogens (*Bacteroides*, *Enterobacter, Enterococcus* and *Helicobacter*). Transcriptomic and molecular analyses revealed that GFXD inhibited hyperactivation of Toll-Imd pathways and JAK-STAT signaling. Finally, three compounds of GFXD, formononetin, kaempferol, and ergosterol were found to alleviate CPT-11 induced intestinal injury.

**Conclusions:**

GFXD alleviates CPT-11-induced intestinal mucositis by modulating gut microbiota composition, suppressing JAK-STAT and Toll-Imd pathways. Thus, this study demonstrates GFXD and its bioactive constituents as novel therapeutic agents to mitigate CIM.

**Graphical Abstract:**

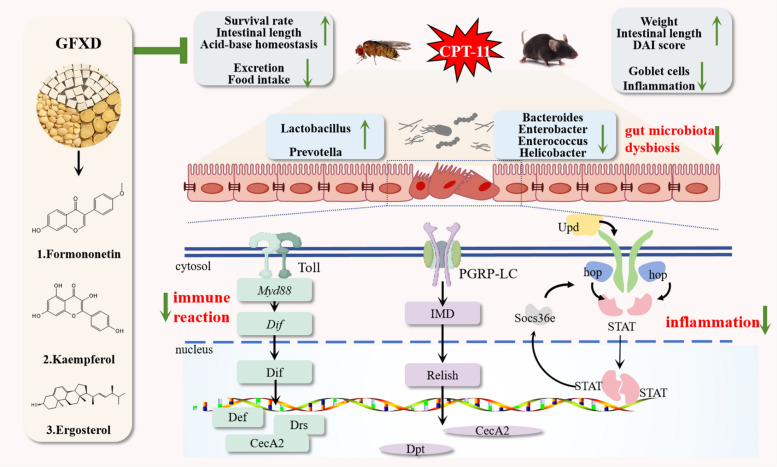

## Highlights

1. GFXD alleviates CPT-11-induced intestinal mucositis in flies and mice by improving survival, enhancing gut integrity and reducing inflammation.
2. GFXD exerts its protective effect by restoring gut microbiota balance and suppressing the hyperactivated JAK-STAT and Toll-Imd immune pathways.
3. The key active compounds Formononetin, Kaempferol and Ergosterol were identified as critical components responsible for GFXD's protective effects against mucositis.

## Introduction

Chemotherapy is a common and fundamental modality for cancer treatment [1]. However, chemotherapy-induced adverse effects encompass a broad spectrum of systemic and organ-specific toxicities, such as gastrointestinal reactions, neurotoxicity, nephrotoxicity, and hepatotoxicity [2, 3]. Chemotherapy-induced intestinal mucositis (CIM) is one of the major adverse effects, and it can compromise both the quality of life and long-term survival rates in cancer patients. Irinotecan (CPT-11) is a chemotherapy agent widely used in clinical practice, but about 40%-80% of patients receiving CPT-11 chemotherapy develop intestinal mucositis [4, 5], which manifests as severe diarrhea, abdominal pain, nausea, and vomiting [6, 7]. Presently, there is a deficiency of validated agents for improving CPT-11 induced CIM in clinical applications. Therefore, it is of great clinical significance to investigate novel agents for alleviating CIM.

The pathogenesis of CIM is multifactorial, involving complex pathophysiological processes, such as inflammation, oxidative stress-mediated tissue damage [8], aberrant immune responses [9], and gut microbiota dysbiosis [10]. Accumulating evidence reveals the pivotal role of gut microbial dysbiosis in modulating CIM pathogenesis primarily via damaging intestinal barrier integrity and disrupting mucosal immune homeostasis. Previous studies have shown that CPT-11 could increase the proportion of pathobionts (e.g., *Desulfovibrio*, *Shigella* and *Helicobacter*) and decrease the beneficial bacteria (e.g., *Lactobacillus_reuteri* and *Akkermansia_muciniphila*), thereby inducing intestinal dysbiosis and exacerbating CIM [11]. Furthermore, intestinal immune function is crucial for preserving intestine against infection by external pathogens. Gut microbiota dysbiosis contributes to chronic immune activation, process to a certain extent by activating the Toll pathway. In addition, inflammation serves as a critical driver of CPT-11-induced intestinal damage, activating the JAK/STAT signaling pathway could induce persistent mucosal inflammation and epithelial injury [12]. Notably, pharmacological inhibition of the JAK/STAT pathway synergistically enhances CPT-11's therapeutic potential in colorectal cancer models, demonstrating dual efficacy in suppressing tumor progression and alleviating chemotherapy-induced intestinal toxicity [13].

Dunhuang Medicine, with a millennium-long historical legacy, represents a vital branch of Dunhuang studies. The comprehensive medical system integrates Traditional Chinese Medicine (TCM) with diverse international medical philosophies [14]. Dunhuang Ancient Medical Prescription synthesizes millennia of empirical medical knowledge, which has demonstrated notable therapeutic potential across various diseases [14, 15]. Among these, Dunhuang Gancao Fuling Xingren decoction (GFXD) originated from the *Dunhuangyishu P.3596*, which is composed of *Poria cocos* (Schw.) Wolf, *Glycyrrhiza uralensis* Fisch.ex DC. [Fabaceae] and *Prunus armeniaca* L. [Rosaceae] (Plant names were checked on July 9, 2025, via MPNS (http://mpns.kew.org)), at a ratio of 1:1:1. Traditionally, GFXD targets Chest Impediment (Xiongbi), a syndrome that manifests multisystem pathology, primarily affecting respiratory, cardiovascular, digestive, and neurological systems [16]. GFXD has been clinically employed to treat diverse conditions, including dilated cardiomyopathy (DCM) and chronic obstructive pulmonary disease (COPD), alongside symptoms of spleen deficiency with dampness exuberance such as poor appetite and loose stools [16]. Its therapeutic basis is rooted in the ability to fortify the spleen and replenish qi, supported by pharmacological studies demonstrating anti-inflammatory and metabolic-improving effects [17, 18]. Notably, CIM is also understood in TCM as a manifestation of spleen deficiency with dampness encumbrance, suggesting the potential applicability of GFXD in its management. Previous investigations have demonstrated that *Poria cocos* and its bioactive constituents exhibit protective properties against cisplatin-induced enteric injury via potentially modulating the intestinal microbial ecology and metabolic profiles [19]. Complementary to these findings, the ethanol extract of *Glycyrrhiza uralensis* Fisch. demonstrates protective effects against diarrheal phenotypes in murine models [19]. Notably, *Prunus armeniaca* exhibits potential to enhance digestive function, regulate immune homeostasis, and confer resistance against Aeromonas veronii infection in host organisms [20]. Collectively, these findings validate the therapeutic promise of GFXD for gastrointestinal treatment, thereby necessitating systematic investigation into its precise mechanisms against chemotherapy-induced intestinal mucositis (CIM).

The mouse is a classic model to study the pathogenesis and drug screening of CIM, and has similar intestinal function with human. In addition, *Drosophila melanogaster* (fruit fly) has been used as a drug-screening model due to its complete genome sequencing and ease of genetic manipulation. In our previous studies, intestinal injury induced by cytarabine (Ara-C) and irinotecan (CPT-11) was investigated using adult flies [21, 22]. The mouse and fly models combine the advantages of high efficiency and precision, which could facilitate the study of the efficacy and mechanism of natural herbs. For example, Astragalus membranaceus-Pueraria lobata could alleviate CIM by inhibiting oxidative stress and Toll pathway in both fly and mice models [23].

This study utilizes *Drosophila melanogaster* and *C57BL/6 J* murine models to systematically evaluate the therapeutic efficacy and mechanisms of GFXD against CPT-11-induced intestinal mucositis. Firstly, the protective effect of GFXD on physiological and intestinal injury caused by CPT-11 were investigated. Subsequently, the mechanism and bioactive compounds of GFXD in alleviating CPT-11 induced intestinal damage were investigated using transcriptomics, LC–MS and fly experiments. These findings establish a mechanistic foundation for the pharmacological validation and clinical translation of classical Dunhuang medical formulae, bridging empirical knowledge from ancient manuscripts with evidence-based therapeutic development through integrated pharmacological and multi-omics characterization.

## Methods and materials

### Experimental drugs

Irinotecan (CPT-11, #100,286–90-6), Myristic acid (#544–63-8), Palmitic acid (#57–10-3), Ergosterol (#57–87-4), Isoliquiritigenin (#961–29-5), Formononetin (#485–72-3), Kaempferol (#520–18-3), Liquiritin (#551–15-5), Amygdalin (#29,883–15-6), and Ursolic acid (#77–52-1) were acquired from Shanghai Yuanye Bio-Technology Co., Ltd. (Shanghai, China). Bromophenol blue (#SHBN7603), and dihydroethidium (#088M2512V) were supplied from Sigma-Aldrich, while Trypan Blue reagent (Catalog No. C0040) was sourced from Beijing Solarbio Science & Technology Co., Ltd. IL-10 (BY-EM220162), IL-6 (BY-EM220188), IL-1β (BY-EM220174), and TNF-α (BY-EM220852) were procured from Boyan Biotechnology Co., Ltd. (Nanjing, China). For immunofluorescence, 7-AAD and DHE were purchased from Abcam (Cambridgeshire, UK). Based on our previous studies, a concentration of 4 mM CPT-11 was chosen for this study [21].

### Preparation of Dunhuang Gancao Fuling Xingren decoction

*Poria cocos*, *Glycyrrhiza uralensis*, and *Prunus armeniaca* were procured from the Affiliated Hospital of Gansu University of Chinese Medicine. *Gancao Fuling Xingren* decoction (GFXD) aqueous extract was prepared through a standardized decoction protocol. Firstly, all herbs were soaked for 30 min with 1 L of ultrapure water and thermal extraction cycles with medium fire for 30 min, which were repeated twice. Pooling of filtrates followed by rotary evaporation under reduced pressure (60℃, 0.08 MPa), and lyophilized into powder at -80 ℃. The extract was stored at -20 ℃.

### Fly stock and experiment

Drosophila melanogaster stocks were maintained under controlled conditions (25 ± 0.5 °C, 65% RH) using standardized maize meal-agar substrate, with photoperiod regimen consisting of 12-h light/dark cycles. The *Esg-Gal4;UAS-GFP*, *Myo1A-GAL4;UAS-GFP*, and *10* × *STAT92E-GFP* flies were used [14, 15, 24]. The wild-type *w*^*1118*^ (#5905) flies were obtained from the Bloomington *Drosophila* Stock Center (BDSC; Indiana, USA).

Adult flies (3–5 days old) of both sexes were randomly assigned to groups to account for potential sex-dependent responses, including the control group, model group (4 mM CPT-11 treatment), different dose of GFXD groups (4 mM CPT-11 with 10, 20 or 40 mg/mL GFXD, respectively).

### Survival rate

20 male or female flies per vial were received in standard cornmeal diet with or without CPT-11 containing GFXD at 10, 20 or 40 mg/mL, individually. The number of dead flies was recorded once a day, and the fresh medium was replaced every 3 d until all flies died. Survival curves were plotted.

### Body weight and starvation assays

Each group of flies on day 9 of the experiment were weighed, and the average weight of each group was calculated. Then flies were transferred to the vial containing 1% agarose for detecting starvation resistance. The number of dead flies was recorded every 6 h until all flies died.

### Intestinal length, ovary and crop size assays

The guts, ovaries and crops were dissected in cold 1 × PBS solution, and placed on a glass slide. The intestinal length was measured and recorded under a microscope with a vernier caliper. The ovary and crop were imaged under a stereomicroscope and the size was measured with Image J.

### Abdominal score assay

15 flies per vial were starved for 6 h and fed with a 2% Bromophenol blue-supplemented diet for 4 h. The abdominal color of each fly was quantification and scored under a light microscope as previously describe [22]

### Excretion assay

10 flies per vial were starved for 6 h, then transferred to a 2 mL centrifuge tube containing small holes in the tube wall and fed food containing 2% Bromophenol blue sodium for 12 h. The faeces were resolved in 600 μl Phosphate-buffered Saline (PBS) and measured at 625 nm [21].

### Intestinal acid–base homeostasis assay

The intestinal acid–base homeostasis assay was used to assess the function of the copper cell region (CCR) [25]. Briefly, 10 flies per vial were starved for 6 h and placed in experimental vial containing 1% agar and 1% bromophenol blue for 4 h. Then, the difference in midgut color was quantified chromatic variations.

### Trypan blue exclusion assay

Trypan blue exclusion testing was performed as previously described [26]. In brief, the intestines of flies were stained in 0.1% trypan blue for 30 min, washed in cold PBS for 3 times, and scored for dark blue staining of midgut. Intestinal cell death was quantified by Trypan blue uptake, with staining intensity scored as: 0 (no color), 1 (light blue), 2 (blue), 3 (dark blue) [14].

### Immunostaining of adult fly guts

Adult *Esg-GAL4;UAS-GFP*; *Myo1A-GAL4;UAS-GFP* and *10* × *STAT-GFP* female flies were dissected in cold PBS. The excised intestinal tissues were fixed with 4% paraformaldehyde for 30 min and then rinsed with PBS for 10 min. In addition, dead cells were detected by 7-amino actinomycin D (7-AAD, Thermo Fisher Scientific). Briefly, each group of flies was dissected in cold PBS, incubated in 7-AAD (5 ug/ml) for 30 min, and rinsed with PBS. The guts were fixed in 4% paraformaldehyde for 30 min. Subsequently, the tissues were observed under an Leica Stellaris 5 confocal laser scanning microscope (Leica, Germany). Images were processed using Image J software.

### Transcriptome analysis

Total RNA was isolated using Trizol reagent following the manufacturer's procedure. Correlation analysis and principal component analysis (PCA) were conducted using princomp function in R (http://www.r- project.org/). Gene expression differences between groups were assessed with the DESeq2 framework. Genes displaying a false discovery rate (FDR) < 0.05 and absolute fold change ≥ 2 were defined as differentially expressed genes (DEGs). These DEGs subsequently underwent systematic functional enrichment analysis for Gene Ontology (GO) terms and KEGG pathways.

### Fecal microbiota transplantation (FMT) experiment

Fecal microbiota transplantation (FMT) experiment was performed according to an established protocol [27]. After all groups were firstly fed with 4 mM CPT-11 in standard food, the flies were cultured on the “dirty medium fed with control group flies” in the FMT-Control group, the flies were cultured on the “dirty medium fed with model group flies” in the FMT-Model group, and the flies were cultured on the “dirty medium fed with GFXD group flies” in the FMT-GFXD group. A separate control group of flies was maintained on 4 mM CPT-11, and survival was monitored daily until complete mortality.

### Antibiotic treatment

For antibiotic treatment, an antibiotic cocktail (150 mg/mL carbenicillin, 150 mg/mL metronidazole, and 75 mg/mL tetracyclin) were added to the food [28]. 3–5 day old females were randomly divided into 6 different groups: control group (standard food), model group (4 mM CPT-11), GFXD group (CPT-11 + 20 mg/mL GFXD), ab-Control group (standard food + antibiotic cocktail), ab-Model group (CPT-11 + antibiotic cocktail), and ab-GFXD group (CPT-11 + GFXD + antibiotic cocktail).

### Quantitative real-time PCR

Approximately 60 guts per group were collected. Total RNA was extracted using Trizol reagent, followed by cDNA synthesis with HiScript® II Reverse Transcriptase (YEASEN, China). Quantitative PCR (qPCR) was performed on a StepOnePlus™ system (Bio-Rad, USA) with SYBR Green Master Mix (Shanghai YEASEN, China) according to manufacturer protocols. The 2^−ΔΔCt^ method was normalized to rp49 as the endogenous control. Primer sequences are detailed in Table 1.

**Table 1:** Primers for qRT-PCR analysis of genes

Genes	Forward	Reverse
*PGRP-LC*	AAACGATCCGTTGACTGGAC	TACGCTTGGATTCCGTTTTC
*Imd*	TTCGGCTCCGTCTACAACTT	GTGATCGATTATGGCCTGGT
*Relish*	ACAGCTACAGGAACTGCATCAGGAA	TCATCCTCCTCGAAGAACCTCACT
*PGRP-SD*	ACTTGGATCGGTTTGCTCATC	AGGGAGTTTCCATGCTGTCTAT
*Myd88*	GGCTCGTTCCCTACACGATC	GAATGCTGGGAGTGGTCACC
*upd2*	CGGAACATCACGATGAGCGAAT	TCGGCAGGAACTTGTACTCG
*upd3*	GAGCACCAAGACTCTGGACA	CCAGTGCAACTTGATGTTGC
*hop*	GTGGGCTCCAAGATACG	GGCAGATACTGAACGGTG
*STAT92E*	CTGGGCATTCACAACAATCCAC	GTATTGCGCGTAACGAACCG
*Socs36E*	CAGTCAGCAATATGTTGTCG	ACTTGCAGCATCGTCGCTTC
*rp49*	CTTCATCCGCCACCAGTC	GCACCAGGAACTTCTTGAATC

### Mouse experiment

Female *C57BL/6 J* mice (18 − 20 g) were acquired from SPF Biotechnology Co., Ltd. (Beijing). Animals were acclimatized for 1 week in a specific pathogen-free (SPF) facility maintained at 21 ± 1℃, 55 ± 5% relative humidity, with a 12 h light/dark cycle. All protocols complied with the NIH Guidelines for Laboratory Animal Care and were approved by the Animal Ethics Committee of Gansu University of Chinese Medicine (Approval No. SY2024-266).

24 mice were randomly allocated into three groups: control, model, and GFXD groups. According to the ratio of the body surface area between humans and mice, it is obtained that the equivalent dosing amount for mice is 9.1 g·kg^−1^·d^−1^. The model and GFXD groups received intraperitoneal injection of CPT-11 at a dose of 75 mg·kg^−1^·d^−1^ for 4 consecutive days. Concurrently, the mice in the GFXD-treated group received 9.1 g·kg^−1^·d^−1^ of GFXD solution via oral gavage for 7 consecutive days. Both control and model groups received equivalent volumes of saline throughout the experimental period.

### Pathological examination in mice

Colonic segments were flushed in PBS, dissected, and immersion-fixed in 4% paraformaldehyde. Following fixation, tissues underwent gradient ethanol dehydration and paraffin embedding. Serial Sects. (5 μm thickness) of the colon were prepared for histological analysis. Tissue slices were sequentially stained with hematoxylin and eosin (H&E) and Alcian blue-periodic acid Schiff (AB-PAS) to evaluate mucosal architecture and mucin content. Morphometric assessments were conducted in triplicate per animal, with final values representing mean measurements.

### Elisa analysis

The intestinal concentrations of IL-10, IL-6, IL-1β, and TNF-α were quantified using commercial ELISA kits (IL-10: BY-EM220162; IL-6: BY-EM220188; IL-1β: BY-EM220174; TNF-α: BY-EM220852) according to the manufacturers' instructions.

### Immunofluorescence staining

Slices were deparaffinized with xylene I and II for 20 min each, anhydrous ethanol I and II for 5 min each, and 75% alcohol for 5 min before washing with water; High pressure antigen repair for 2.5 min, washed three times with PBS; Inactivate with 0.3% methanol and hydrogen peroxide for 15 min, wash with PBS three times; Organize pen circle tissue, block with 5% BSA for 1 h; add PBS diluted primary antibody (Occludin 1:400, ZO-1 1:200, JAK1 1:300, STAT3 1:300) dropwise for incubation, wash with PBS three times (8 min each); Incubate with HRP secondary antibody for 50 min, wash with PBS three times; Add corresponding fluorescent TSA dropwise and incubate in the dark for 10 min, wash with PBS three times; Incubate with DAPI for 10 min, wash with PBS 3 times; Anti fluorescence quenching agent sealing, fluorescence microscope observation and photography.

### 16 s rRNA sequencing analysis of faecal DNA extracts

Fecal genomic DNA was isolated, and the bacterial 16S rRNA V3–V4 hypervariable region was amplified via PCR using universal primers 338F (5’-ACTCCTACGGGAGGCAGCA-3’) and 806R (5’-GGACTACHVGGGTWTCTAAT-3’) as described [29]. Next, amplification products were purified with Vazyme VAHTSTM DNA Clean Beads (Nanjing, China), and DNA concentrations were determined using the Quant-iT PicoGreen dsDNA Assay Kit (Invitrogen, USA). Equimolar amplicon pools were constructed and subjected to paired-end sequencing (2 × 250 bp) on an Illumina MiSeq platform at Shanghai Personal Biotechnology Co., Ltd.

### LC–MS analysis of GFXD components

GFXD powder was lyophilized (vacuum freeze-drying), pulverized, and homogenized. Aliquots (50 mg) were vortex-mixed with 1200 μL of pre-cooled 70% methanol, followed by centrifugation (12,000 rpm, 3 min). The resultant supernatant was filtered through a 0.22 μm microporous membrane for LC–MS analysis. Ultra-high-performance liquid chromatography (UHPLC; ExionLC™ AD, Sciex) coupled with tandem mass spectrometry (QTRAP^®^ 6500, Sciex) was conducted by Metware Biotechnology Co., Ltd. Targeted multiple reaction monitoring (MRM) transitions were dynamically selected based on the chromatographic elution profile of metabolites [30].

### Statistical analysis

Data were analyzed by GraphPad Prismversion 9.0 software and were presented as mean values ± standard error of mean values (S.E.M). The Log-rank test was used to determine the statistical significance of survivorships among the groups. One-way ANOVA test was performed on the statistical significance among the different experimental groups. Unpaired Student’s T-test was performed to analyze the statistical significance between two groups. The significance level was indicated as #*P* < 0.05, ##*P* < 0.01, ###*P* < 0.001, compared with the control group; *P < 0.05, **P < 0.01 and ***P < 0.001, compared with the model group.

## Results

### GFXD ameliorates the physiological damage induced by CPT-11 in flies

**Fig. 1:**
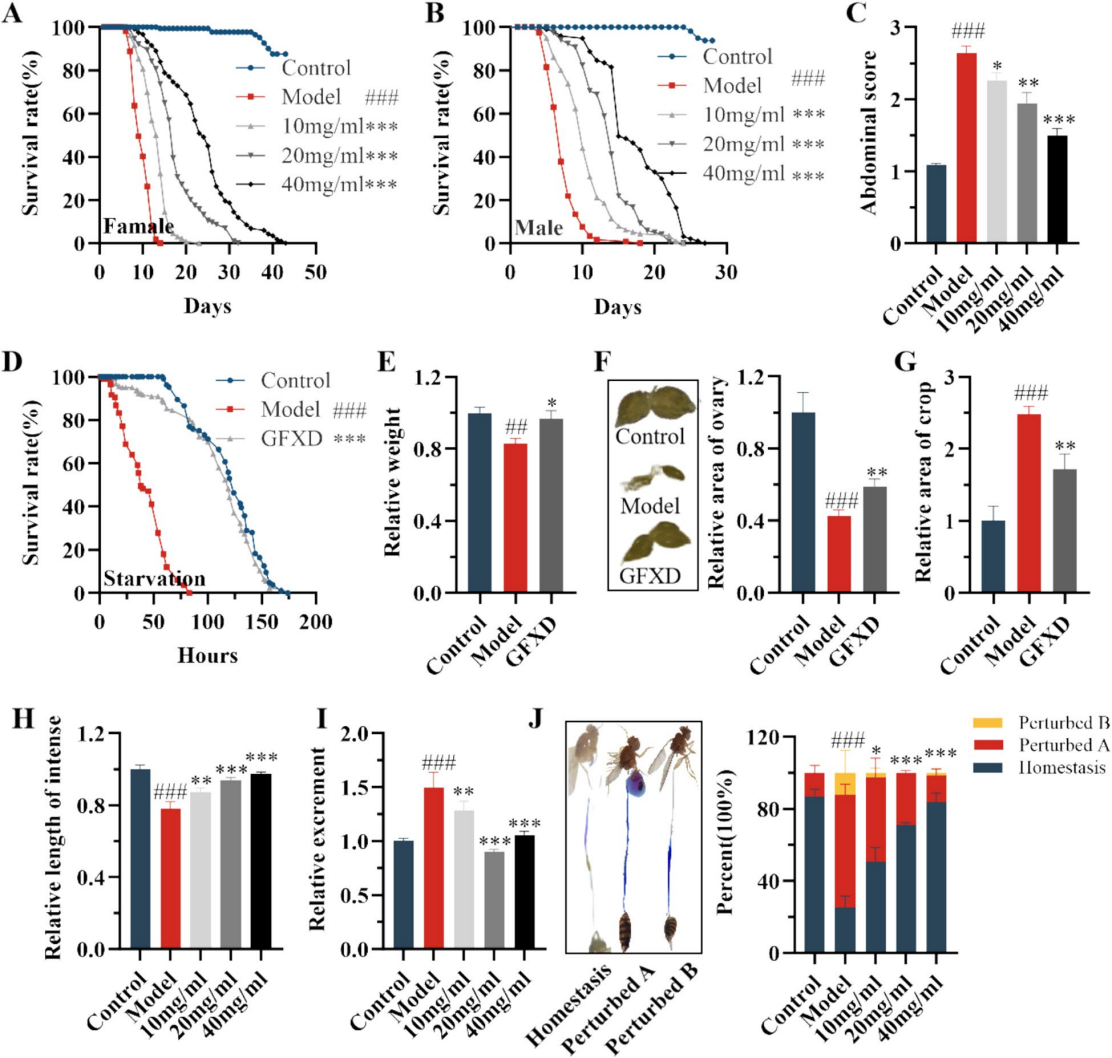
GFXD alleviates CPT-11-induced physiological and intestinal damage in flies. The survival rate of adult female (**A**) and male (**B**) flies (n = 100–110). (**C**) Abdominal scores (n = 6). (**D**) Starvation resistance (n = 6). (**E**) Relative weight of flies (n = 6). **F** Schematic diagram of ovarian morphology and the relative area of ovary. **G** Size of crop (n = 6). **H** Length of intestine (n = 12). **I** The excretion of flies (n = 6). **J** Schematic diagram of PH indicator in the gastrointestinal tract and quantification of the percentage of acid–base homeostasis (n = 6). Data are presented as mean ± SEM. ##*P* < 0.01, ###*P* < 0.001, compared with the control group; *P < 0.05, **P < 0.01 and ***P < 0.001, compared with the model group

To determine whether GFXD has a protective effect on CPT-11 induced physiological damage, the survival rate, food intake, starvation resistance, weight and ovarian morphology were determined in adult flies. Administration of GFXD could increased survival rates in both female (Fig. 1A) and male (Fig. 1B) flies. Meanwhile, GFXD supplementation remarkably decreased the food consumption in CPT-11 treated flies, which was dose dependent (Fig. 1C).

Since the middle dose of GFXD showed significant protective effects, 20 mg/mL GFXD was selected for further experiment. Administration of GFXD at 20 mg/mL could significantly enhance the starvation resistance and weight loss in female flies treated with CPT-11 (Fig. 1D and E), which indicated that GFXD alleviated the metabolic damage induced by CPT-11. Meanwhile, GFXD supplementation remarkably restored the ovarian atrophy caused by CPT-11 (Fig. 1F), indicating that GFXD alleviated the reproductive capacity damage induced by CPT-11. Taken together, GFXD has ability to alleviate CPT-11 induced body injury in adult flies.

### GFXD restores CPT-11 induced intestinal injury in flies

The clinical utility of CPT-11 is limited by delayed-onset diarrhea and associated intestinal mucosal damage. To determine whether GFXD could alleviate the CPT-11 induced intestinal injury, the excretion, length of intestine, area of crop, gastrointestinal acid–base homeostasis were measured in females. Supplementation of GFXD remarkably decreased the excretion in flies fed with CPT-11 (Fig. 1I). Meanwhile, the gastrointestinal acid–base homeostasis, area of crop and the length of intestine were also examined. Administration of GFXD significantly rescued the shorten of intestinal length and inhibited the increased size of the crop in CPT-11 treated flies (Fig. 1G and H), suggesting that GFXD could alleviate the disruption of gastrointestinal morphology. Additionally, intestinal acid-based homeostasis was disrupted in model group, whereas supplementation of GFXD could restore the imbalance in CPT-11 treated flies (Fig. 1J). Therefore, GFXD inhibits the CPT-11 induced intestinal mucocitis.

### GFXD rescues CPT-11 induced intestinal cell damage

**Fig. 2:**
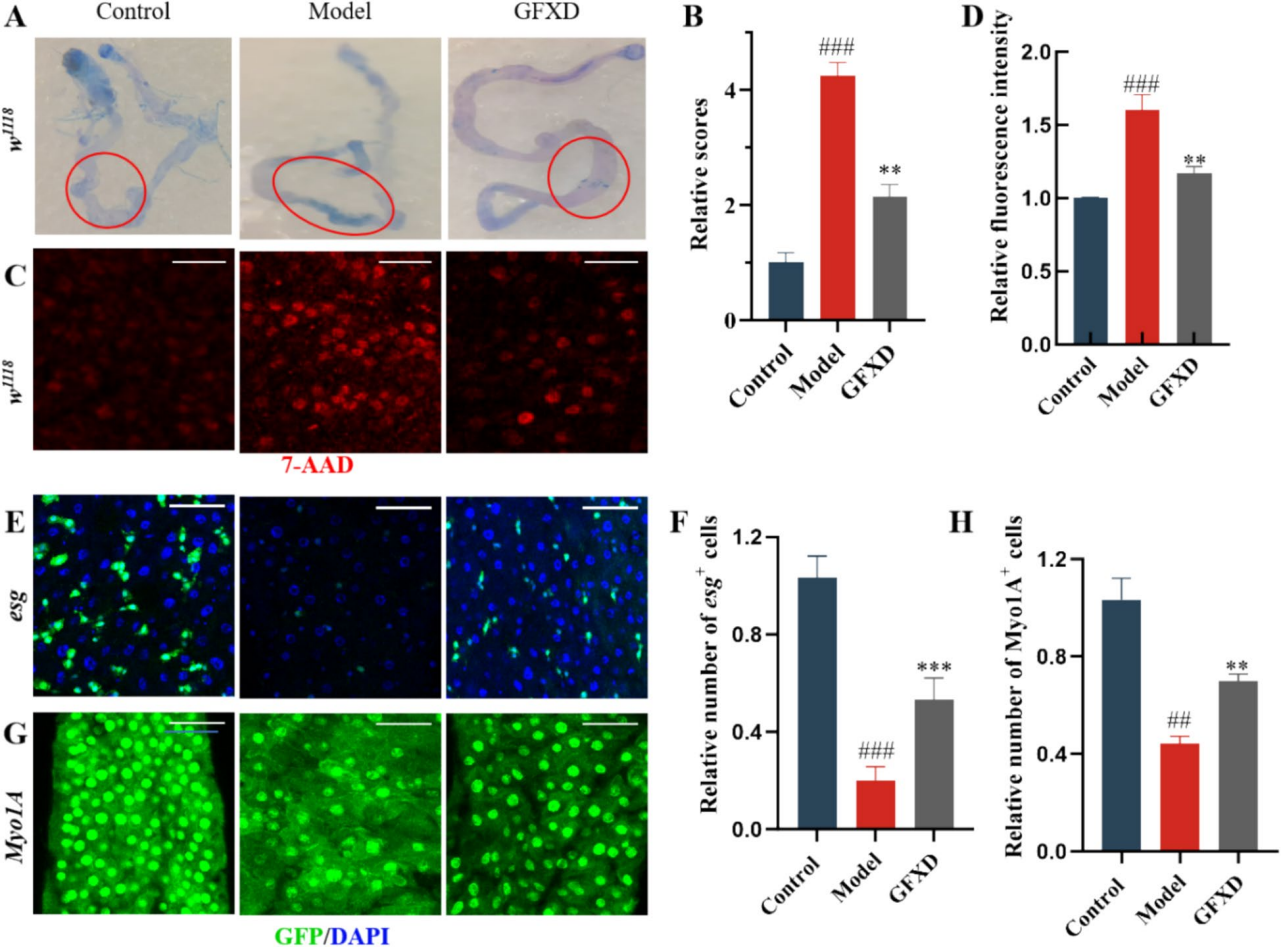
GFXD alleviates CPT-11-induced intestinal epithelial cell damage in flies. **A**–**B** Trypan blue stains scoring schematic and quantitative statistics (n = 6). **C**-**D** 7-AAD stains scoring schematic and quantitative statistics (n = 6). **E**–**F** Intestinal *Esg-GFP* staining schematic and quantitative statistics (n = 6). **G**-**H** Intestinal *Myo1A-GFP* staining schematic and quantitative statistics (n = 6). (Bar = 100 μm). Data are presented as mean ± SEM. ##*P* < 0.01, ###*P* < 0.001, compared with the control group; **P < 0.01 and ***P < 0.001, compared with the model group

Death of intestinal cells is one reason for intestinal injury, which could be detected by trypan blue exclusion testing and 7-AAD staining. The result revealed that GFXD could significantly reduce dead cells in the midguts of CPT-11-induced flies (Fig. 2A and B). This was corroborated by decreased 7-AAD⁺ cells in GFXD-supplemented flies versus CPT-11 controls (Fig. 2C and D). *Myo1A-GAL4;UAS-GFP* flies were used to label intestinal epithelial cells (IECs), we observed that CPT-11 severely depleted IECs, while GFXD restored their numbers (Fig. 2G and H). Previous studies showed that CPT-11 not only disrupted epithelial integrity, but also reduced the regenerative capacity of the gut, specifically affecting the proliferation capacity of intestinal stem cells (ISCs) [31, 32]. Quantification of ISCs (*Esg-GAL4;UAS-GFP*) confirmed CPT-11-induced ISC depletion, which was rescued by GFXD (Fig. 2E and F). Collectively, GFXD mitigates CPT-11-induced intestinal cytotoxicity and preserves ISC populations.

### GFXD alters CPT-11 induced genetic changes in the gut of flies

**Fig. 3:**
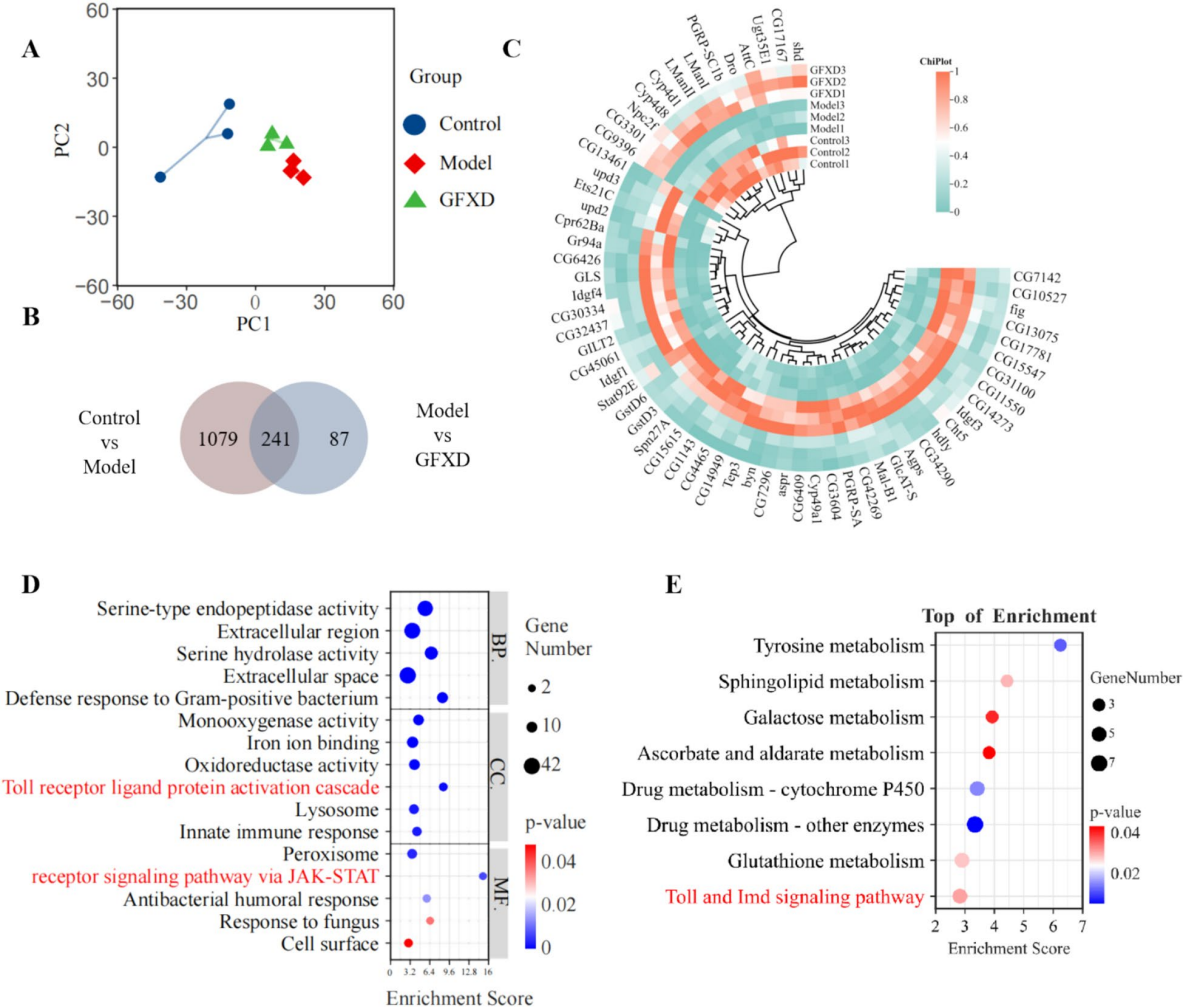
Transcriptome analysis and pathway enrichment of differential expressed genes in intestinal tissues of flies (n = 3). **A** Principal component analysis (PCA) between the control, model, and GFXD groups. **B** Venn plot indicated the screening of differential regulated genes in model after GFXD treatment. **C** Cluster analysis was performed on the significant (*P* < 0.01) DEGs identified. Enrichment analysis of potential GFXD targets by GO (**D**) and KEGG (**E**) analysis

To further investigate the protective mechanism of GFXD against CIM, transcriptomics analysis was used. The analysis of principal component analysis (PCA) showed that the change of gene expression in model group was significantly recovered after the treatment of GFXD (Fig. 3A). Differentially expressed genes (DEGs) were identified by pairwise comparison of groups (FC > 2 or less than 0.5, p.adj < 0.05). There were 1,320 different genes between model and control groups, and 431 different genes between GFXD treatment and model groups (Fig. 3B). The significantly enriched genes were intersected, yielding 241 common genes. GFXD may alleviate CPT-11-induced intestinal injury by regulating these genes (Fig. 3C). GO (Fig. 3D) and KEGG (Fig. 3E) enrichment of the above genes revealed that the therapeutic effect of GFXD against CIM may be mainly related to the defence response to flora and inflammatory response, and closely related to the Toll-Imd signaling pathway as well as JAK-STAT pathway.

### GFXD inhibits JAK-STAT signaling pathway in intestine

**Fig. 4:**
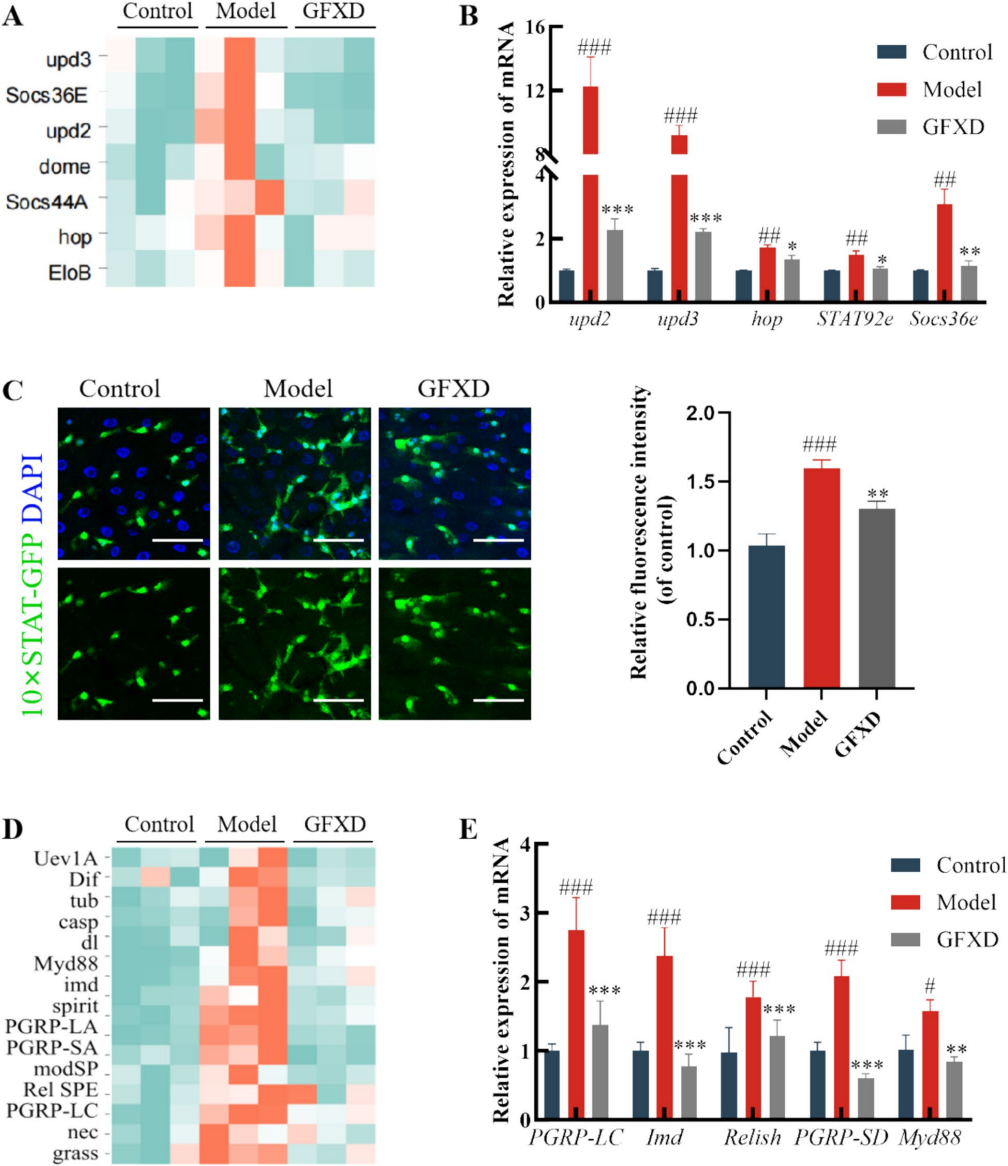
GFXD attenuated CPT-11-induced immune and inflammatory reaction response in flies. **A** Heatmap of JAK-STAT pathway by transcriptome analysis. **B** The gene expressions of JAK-STAT pathway (*upd2*, *upd3*, *hop*, *STAT92e* and *Socs36e*) were verified by qRT-PCR (n = 6). **C** Immunostaining of the midgut of *10* × *STAT-GFP* staining schematic and quantitative statistics (n = 6). **D** Heatmap of Toll-Imd pathway by transcriptome analysis. **E** The gene expressions of Toll (*Myd88, Dif, and Drs*) and Imd (*PGRP-LC, Imd, and Relish*) pathways was verified by qRT-PCR (n = 6). Data are presented as mean ± SEM. #*P* < 0.05, ##*P* < 0.01 and ###*P* < 0.001, compared with the control group; *P < 0.05, **P < 0.01 and ***P < 0.001, compared with the model group

The activation of JAK-STAT pathway has a certain correlation with the imbalanced gut microbiota [33]. The gene expressions of the JAK-STAT signalling pathway was demonstrated in a heatmap based on the results of the transcriptome analysis, in which GFXD treatment significantly restored the expression of these genes (Fig. 4A). Next, the expression of the JAK/STAT pathway-related genes, such as *upd2*, *upd3*, *hop*, *STAT92e* and *Socs36e* were determined using the qRT-PCR. Supplementation of GFXD remarkably decreased the mRNA expression levels of these genes in CPT-11 treated flies (Fig. 4B). STAT activation was monitored using *10* × *STAT-GFP* reporter flies. CPT-11 exposure significantly increased GFP⁺ cell numbers versus controls (Fig. 4C), whereas GFXD administration reduced intestinal GFP⁺ signal (Fig. 4D), indicating attenuated STAT protein levels. Consequently, GFXD inhibits CPT-11-induced JAK/STAT pathway activation in the Drosophila intestine.

### GFXD inhibits Toll-Imd signaling pathways in intestine

Transcriptome analysis revealed significant enrichment of the Toll-Imd signaling pathways were significantly enriched (Fig. 4D). Furthermore, the CPT-11-induced hyperactivation of these immune pathways was significantly suppressed by GFXD treatment. To further verify this result, the gene expression of Toll (*Myd88, Dif, and Drs*) and Imd (*PGRP-LC, Imd, and Relish*) pathways were examined using qRT-PCR (Fig. 4E). The results showed that the expressions of these genes were significantly up-regulated in the CPT-11-treated group compared to the control group (Fig. 4E), while administration of GFXD suppressed these expression levels. Taken together, GFXD inhibits CPT-11-associated immune hyperactivation in the intestine.

### GFXD alleviates the intestinal injury by regulating the balance of gut microbiota in CIM flies

The gut microbiota serves as a key regulator for the host to maintain intestinal health and the integrity of intestinal immunity. Notably, it has been found that abnormal activation of the immune system such as Toll-Imd signaling pathways is tightly linked to the gut microbiota dysbiosis. Using microbiota depletion by administration of antibiotic and fecal microbiota transplantation (FMT), we further demonstrated whether GFXD could treat CIM via regulating the gut microbiota.

**Fig. 5:**
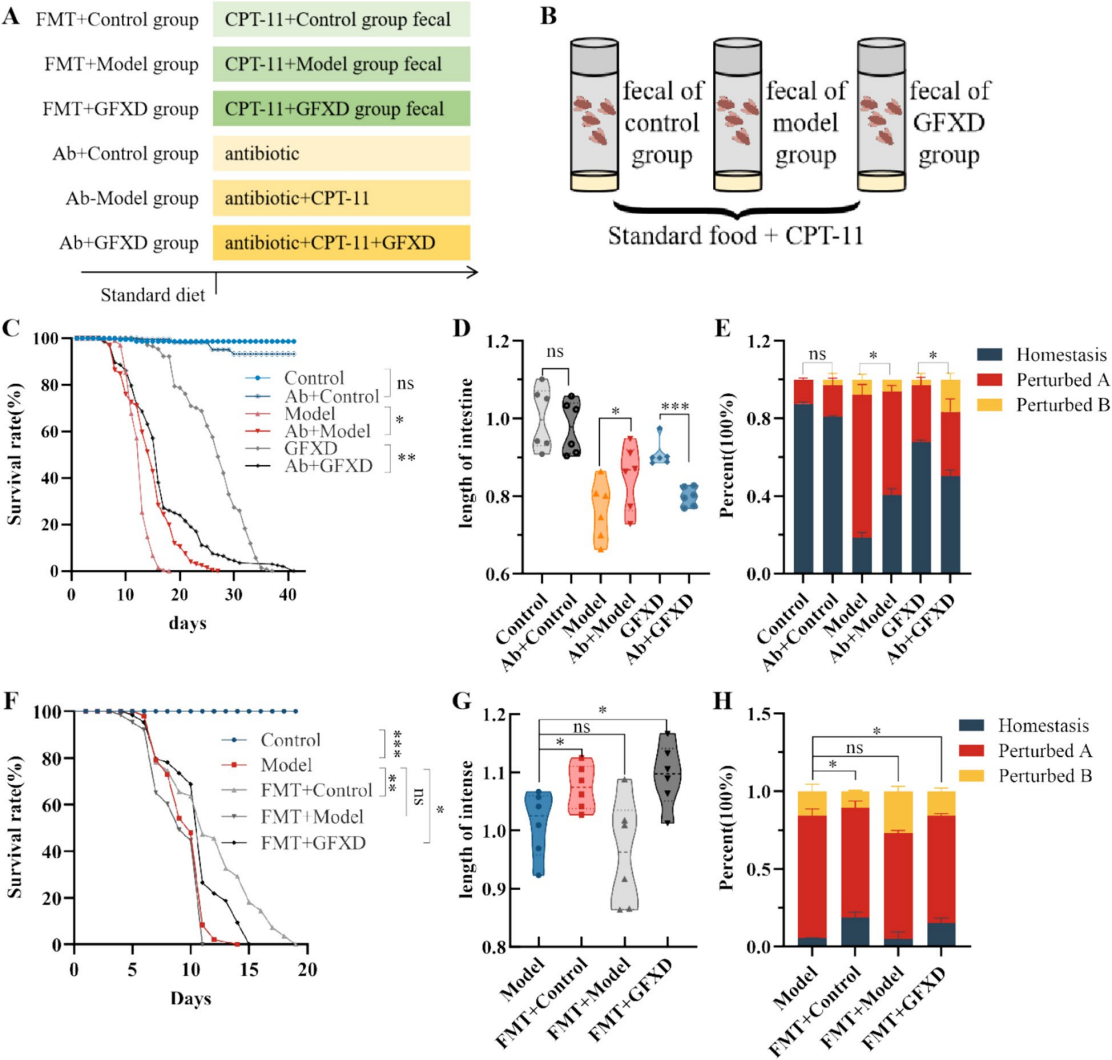
Gut-repairing effect of GFXD achieved via gut microbiota remodeling in flies. **A** Outline of the experimental strategy. **B** Schematic diagram of the FMT experimental procedure. **C** Survival rate (n = 6), **D** length of intestine (n = 6) and (**E**) PH experiment (n = 6) were compared across six experimental groups: Control group, Model group, GFXD group, Ab+Control group, Ab+Model group and Ab+GFXD group. **F** Survival rate (n = 6), (**G**) length of intestine (n = 6) and (**H**) PH experiment (n = 6) were compared across five experimental groups: Control group, Model group, FMT+Control group, FMT+Model group and FMT+GFXD group. Data are presented as mean ± SEM. *P < 0.05, **P < 0.01 and ***P < 0.001

Firstly, the survival rate of flies was increased after treatment with antibiotics in model group, while antibiotics had only a minor effect on the flies in the control group (Fig. 5C). Meanwhile, antibiotics could reduce survival in the GFXD group (Fig. 5C). Similarly, administration of antibiotic significantly rescued the shortening of intestinal length and inhibited the intestinal acid-based homeostasis in CIM flies, whereas supplementation of antibiotics could reduce the efficacy of GFXD in CIM flies (Fig. 5D and E). Secondly, CIM flies fed with fecal from control and GFXD group could prolong the survival rate of the flies, while the FMT-Model did not affect the survival of the flies (Fig. 5F). FMT-Control and FMT-GFXD had increased the length of intestine and restored the intestinal acid-based homeostasis compared to model flies, while FMT-Model flies had the similar intestinal phenotype with model flies (Fig. 5G, H). In conclusion, GFXD alleviates CPT-11 induced intestinal injury via regulating the balance of gut microbiota.

### GFXD ameliorates the physiological and intestinal damage caused by CPT-11 in mice

**Fig. 6:**
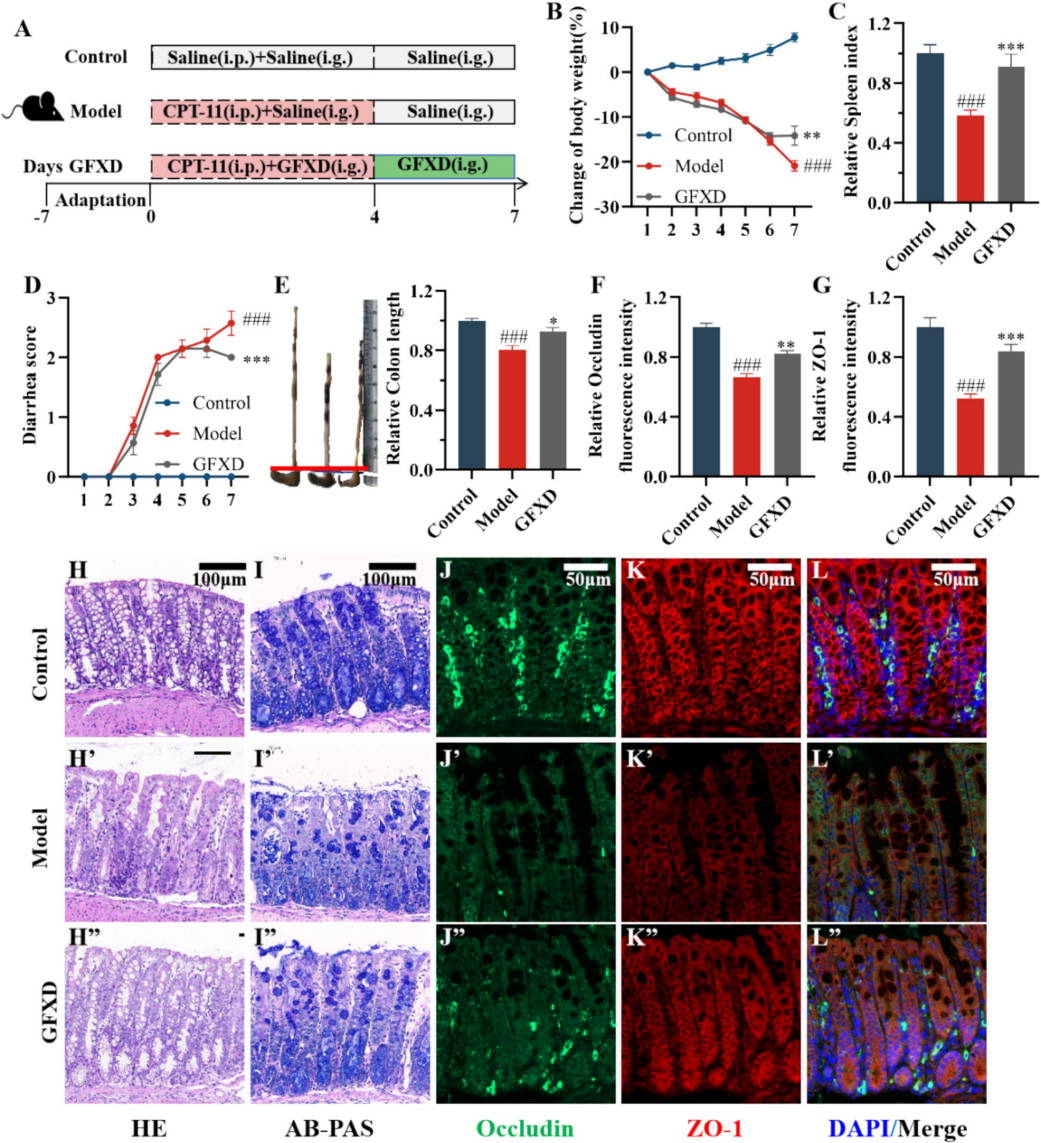
GFXD attenuates CPT-11 induced intestinal mucositis in mice. **A** Outline of the experimental strategy (n = 7). **B** Change of body weight (n = 7). **C** Spleen index (n = 7). **D** Diarrhea score. **E** The length of colon (n = 7). **H** HE staining (n = 3) and (**I**) AB-PAS staining (n = 3) of colon tissue sections (Bar = 100 μm). Expression levels of Occludin (**J**) and ZO-1(**K**) protein. **L** Merged immunofluorescence images. Quantitative analysis of Occludin (**F**) and ZO-1(**G**) protein (n = 3) (Bar = 50 μm). Data are presented as mean ± SEM. #*P* < 0.05, ##*P* < 0.01 and ###*P* < 0.001, compared with the control group; *P < 0.05, **P < 0.01 and ***P < 0.001, compared with the model group

To verify whether the therapeutic effect of GFXD was conserved in mammals, a mouse model of intestinal mucositis was established (Fig. 6A). Compared to the control group, injection of CPT-11 reduced body weight and spleen index in mice. Administration of GFXD significantly restored body weight and spleen index in CPT-11-induced CIM mice (Fig. 6B, C). GFXD treatment also attenuated the elevated disease activity index (DAI) scores (Fig. 6D) and prevented CPT-11-induced colon shortening (Fig. 6E).

Colon histopathology assessed by HE staining revealed significant alterations in CPT-11-induced CIM mice compared to controls (Fig. 6H). These included crypt architectural distortion, epithelial barrier disruption, and focal inflammatory cell infiltration. GFXD treatment substantially attenuated these pathological changes, restoring crypt density while reducing immune cell influx. Notably, AB-PAS staining, which was used to detect the goblet cells in colon tissue sections, revealed GFXD replenished goblet cell to corroborate its mucosal repair capacity (Fig. 6I). The expressions of ZO-1 and Occludin proteins in colon tissues were detected by immunofluorescence staining (Fig. 6J-L). Compared with the control group, the expressions of ZO-1 and Occludin proteins in the colonic mucosal epithelial cells of mice in the CIM model group were significantly weakened, and GFXD could reduce the expression levels of ZO-1 and Occludin proteins (Fig. 6F, G). Taken together, GFXD protects against CPT-11-induced intestinal injury in mice.

### GFXD inhibited IL-6/JAK1/STAT3 signaling pathway in colon tissue of CIM mice

**Fig. 7:**
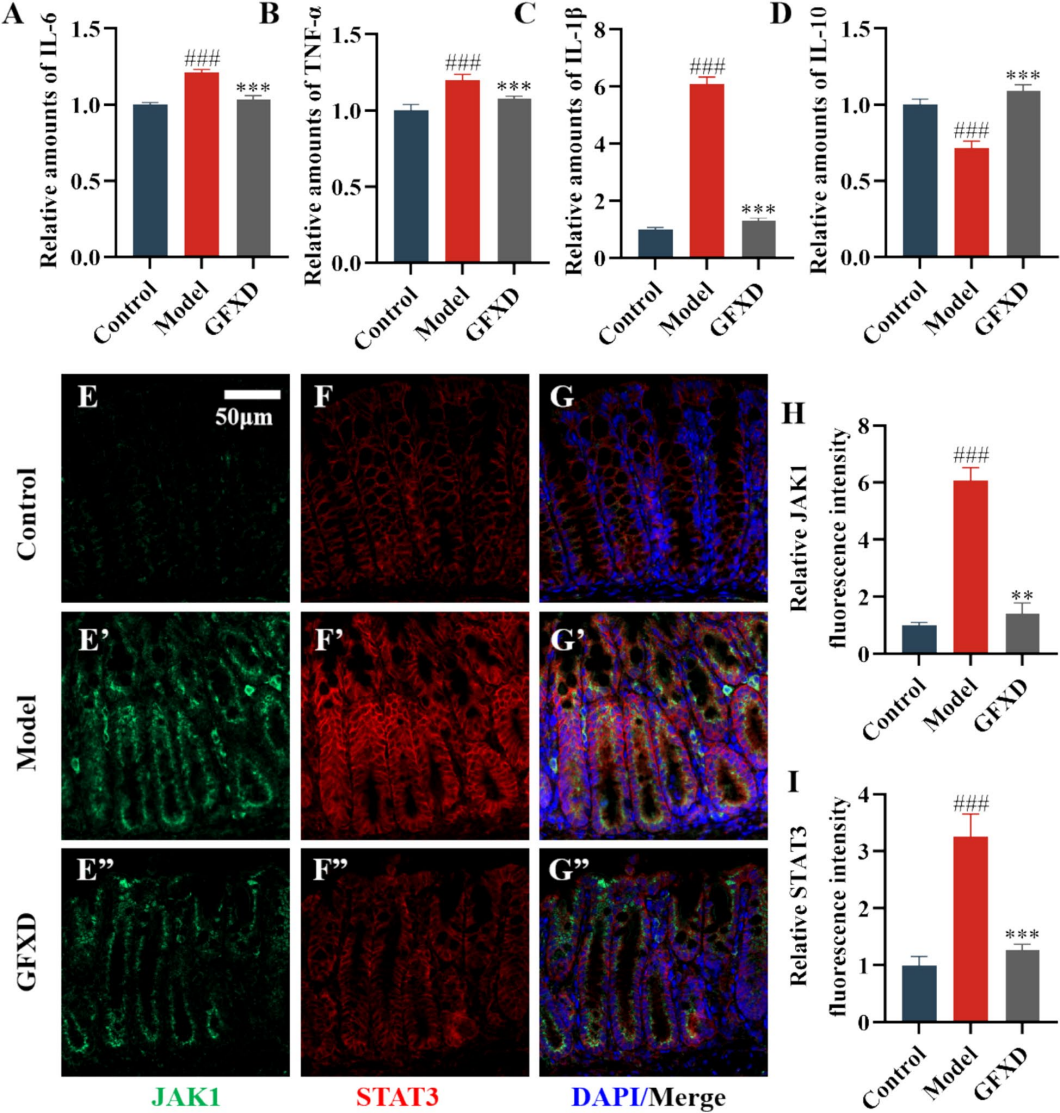
GFXD attenuates IL-6/JAK1/STAT3 signaling pathway in mice. Changes in IL-6 (**A**), TNF-α (**B**), IL-1β (**C**), IL-10 (**D**) levels in intestinal tissues (n = 6). Expression levels of JAK1 (**E**) and STAT3 (**F**) protein. (**G**) Merged immunofluorescence images. Quantitative analysis of JAK1 (**H**) and STAT3 (**I**) protein (n = 3) (Bar = 50 μm). Data are presented as mean ± SEM. #*P* < 0.05, ##*P* < 0.01 and ###*P* < 0.001, compared with the control group; *P < 0.05, **P < 0.01 and ***P < 0.001, compared with the model group

To examine the effect of GFXD on the inflammatory cytokines in CPT-11-treated mice, the contents of IL-6, IL-1β, TNF-α, and IL-10 in different groups of mice were determined. The ELISA findings demonstrated that the level of pro-inflammatory cytokine IL-6, IL-1β, TNF-α elevated, while the anti-inflammatory cytokine IL-10 decreased in model group. GFXD supplementation remarkably restored these abnormal inflammatory cytokine levels (Fig. 7A-D).

As a key biomarker of CIM, inflammatory factors are regulated through multiple pathways. IL-6 is the functional ortholog of Drosophila Upd3 in mice [34]. To further identify the specific JAK/STAT subtypes affected by GFXD, the expression of JAK1 and STAT3 in mouse colon tissue was quantified by immunofluorescence staining. Specifically, as illustrated in Fig. 7E-I, the CPT-11-induced model group exhibited a significant upregulation in the expression and phosphorylation levels of JAK1 and STAT3 proteins in intestinal tissues compared to the control group. Importantly, treatment with GFXD substantially suppressed this activation, leading to a notable downregulation of both total and phosphorylated forms of JAK1 and STAT3. These findings suggest that GFXD exerts its protective effects by specifically inhibiting the activation of the JAK1/STAT3 signaling pathway in the context of CPT-11-induced intestinal injury.

### GFXD intervenes gut microbiota dysbiosis caused by CPT-11 in mice

**Fig. 8:**
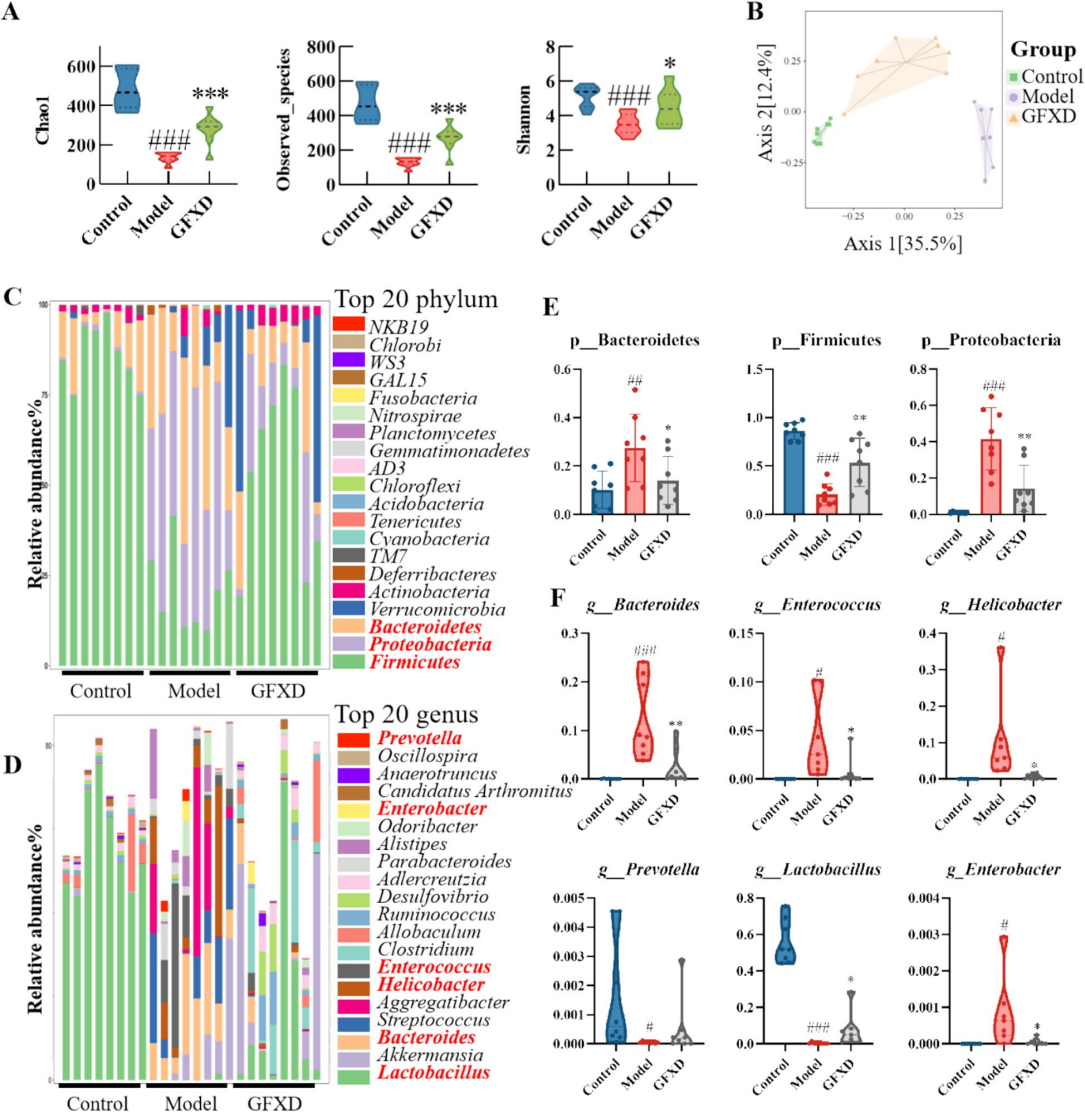
GFXD ameliorates CPT-11 induced intestinal mucositis by modulating gut microbiota in mice (n = 8). **A** Chao1, observed_species and shannon index. **B** Bray–Curtis principal coordinate analysis (PCoA) analysis. Compositions at the phylum (**C**) and genus (**D**) levels. Relative abundance of specific microbiota at the phylum (**E**) level and genus (**F**) level. Data are presented as mean ± SEM. #*P* < 0.05, ##*P* < 0.01 and ###*P* < 0.001, compared with the control group; *P < 0.05, **P < 0.01 and ***P < 0.001, compared with the model group

To further detect the therapeutic role of GFXD via regulating gut microbiota dysbiosis in mammals, we conducted 16S rRNA sequencing of fecal samples [35]. Firstly, GFXD supplementation could restore the reduction of Chao1, Goods_coverage, Observed_species and shannon index in the model group. The results indicate that GFXD supplementation could increase the alpha diversity of the gut microbiota (Fig. 8A). Bray–Curtis principal coordinate analysis (PCoA) revealed that the microbial composition in CPT-11-treated mice was significantly different from that in the control and GFXD groups (Fig. 8B).

To further detect the specific changes of microbial community at the phylum and genus level, the taxonomic composition of samples from each group was analyzed (Fig. 8C-D). At the phylum level, GFXD supplementation remarkably rescued depleted Proteobacteria and Bacteroidetes, and suppressed elevated Firmicutes in CPT-11 treated mice (Fig. 8E). Furthermore, at the genus level, GFXD significantly suppressed opportunistic pathogens linked to inflammation (e.g., *Bacteroides*, *Enterococcus*, and *Helicobacter*) that were elevated by CPT-11, and immune dysregulation (e.g., *Enterobacter*), concurrently promoting the growth of beneficial genera which could contribute to the maintenance of mucosal immune homeostasis, including anti-inflammatory taxa (e.g., *Lactobacillus*) and immune-modulating taxa (e.g., *Prevotella*) (Fig. 8E). Notably, GFXD supplementation ameliorated CPT-11-induced gut microbiota dysbiosis, thereby rescuing the disrupted mucosal immune homeostasis in mice.

### Formononetin, kaempferol and ergosterol as the major compounds of GFXD alleviate intestinal injury caused by CPT-11

**Fig. 9:**
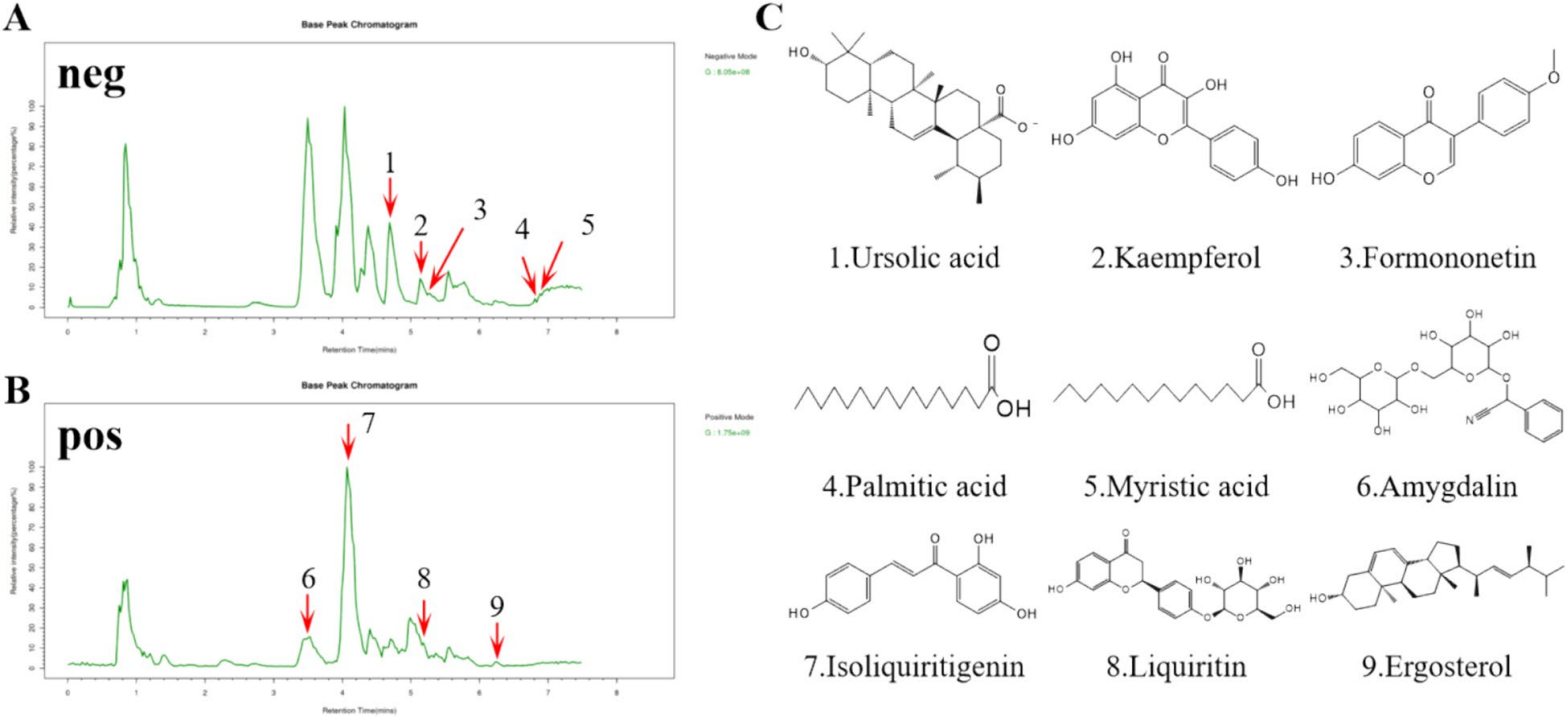
Chemical components in GFXD. **A** negative mode and **B** positive mode of GFXD total ion chromatograms. **C** Nine chemical structures of leading component in GFXD

LC–MS analysis was conducted to determine the bioactive compounds of GFXD against CIM (Fig. 9). According to the results of LC–MS and literature studies, nine bioactive compounds (listed in Table 2) may have therapeutic potential for treating CIM, which were used to detect the protective role under CPT-11 stimulation in flies.

**Table 2:** Chemical components of GFXD

NO	Compounds	MZ	Formula	Class	Ionization model	Peak area
1	Isoliquiritigenin	257.0809	C_15_H_12_O_4_	Flavonoids	[M + H]^+^	68591934420
2	Amygdalin	475.1971	C_20_H_27_NO_11_	Alkaloids	[M + NH_4_]^+^	13018416519
3	Formononetin	267.0659	C_16_H_12_O_4_	Flavonoids	[M-H]^−^	1774959328
4	Palmitic acid	255.2357	C_16_H_32_O_2_	Fatty acids	[M-H]^−^	1604389407
5	Kaempferol	285.0411	C_15_H_10_O_6_	Flavonoids	[M-H]^−^	307730786.5
6	Liquiritin	419.1305	C_21_H_22_O_9_	Flavonoids	[M + H]^+^	36870730.6
7	Ergosterol	353.3527	C_28_H_44_O	Steroids	[M-CO_2_ + H]^+^	12818536.52
8	Myristic acid	227.2017	C_14_H_28_O_2_	Fatty acids	[M-H]^−^	10707128.94
9	Ursolic acid	517.3599	C_30_H_48_O_3_	Terpenoids	[M + HCO_3_]^−^	9162457.328

**Fig. 10:**
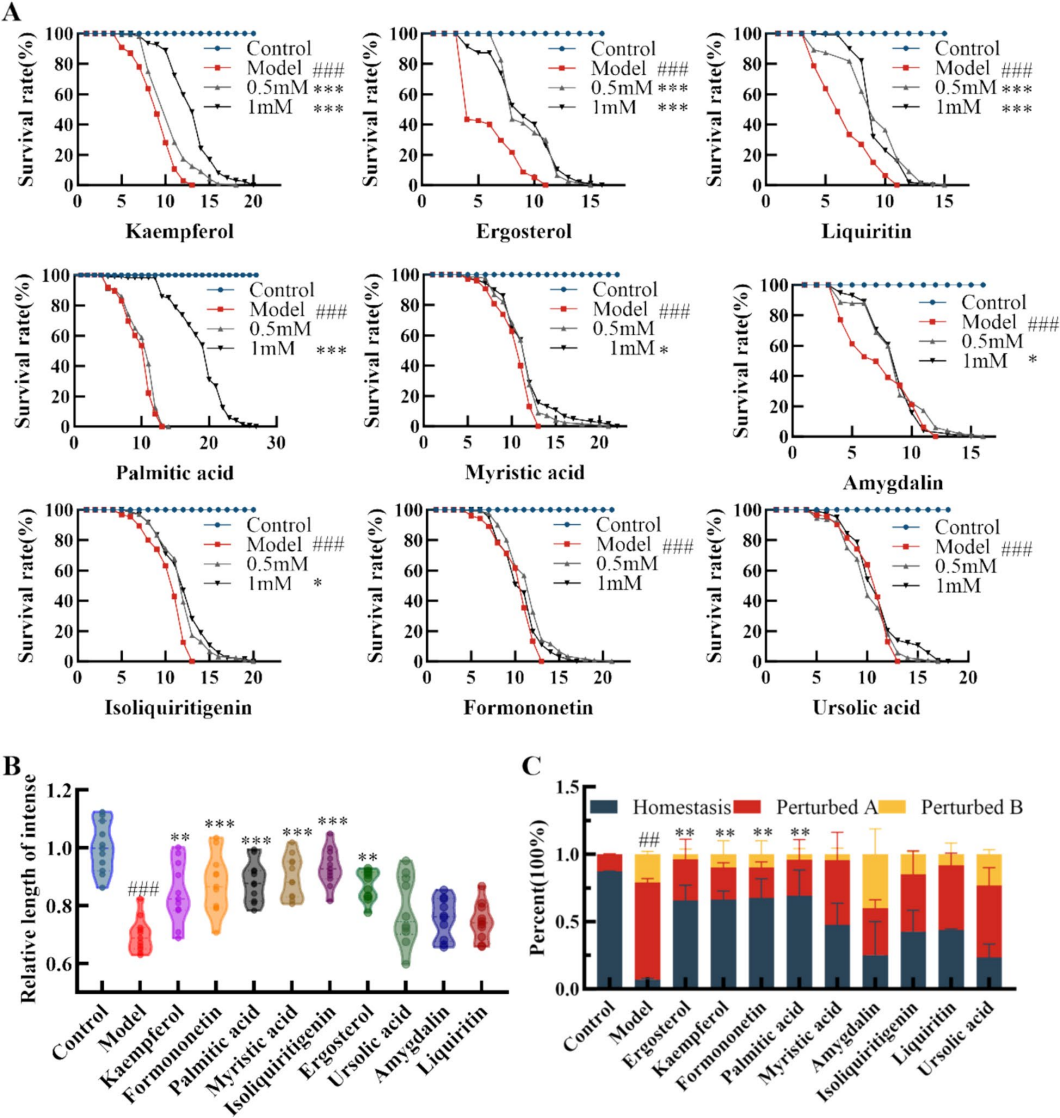
Bioactive compounds in GFXD protect against CPT-11-induced intestinal damage in flies. Effects of active small molecules of GFXD on (**A**) survival rate (n = 80–100), (**B**) intestinal length (n = 6), and (**C**) intestinal homeostasis (n = 6) in CIM flies. Data are presented as mean ± SEM. #*P* < 0.05, ##*P* < 0.01 and ###*P* < 0.001, compared with the control group; *P < 0.05, **P < 0.01 and ***P < 0.001, compared with the model group

The experiments showed that six compounds-kaempferol, ergosterol, liquiritin, amygdalin, isoliquiritigenin, and myristic acid could improve the survival rate in flies fed with CPT-11 (Fig. 10A). Meanwhile, administration of formononetin, palmitic acid, kaempferol, isoliquiritigenin, ergosterol, and myristic acid could recover the shortened intestinal length (Fig. 10B), while formononetin, kaempferol, ergosterol and myristic acid could ameliorate the disrupted intestinal homeostasis in CPT-11-treated flies (Fig. 10C). Taken together, these results indicate that formononetin, kaempferol and ergosterol are the main bioactive compounds of GFXD against CIM.

## Discussion

**Fig. 11:**
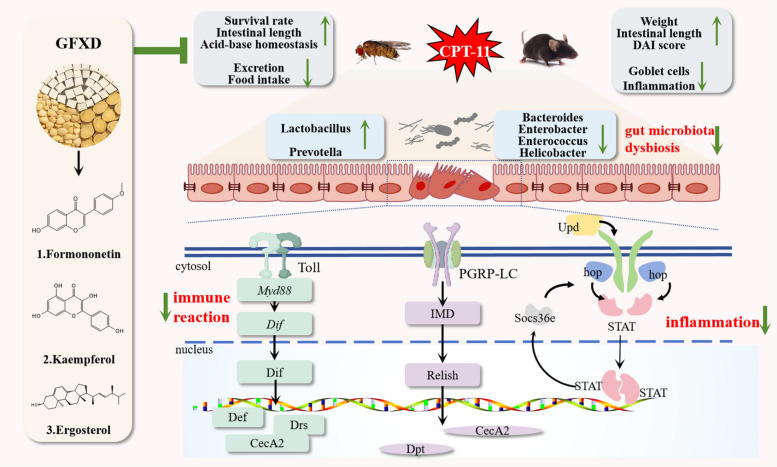
Graphical Abstract. Mechanism of action of GFXD in the treatment of CIM

CPT-11 is used extensively as an anti-cancer agent in the treatment of various tumours, while it seriously affects the treatment process. Therefore, it is necessary to find novel agents to alleviate CIM. Here, we systematically explored the therapeutic effect of GFXD on CPT-11-induced injury, especially intestinal injury. GFXD mainly exerted protective effects by regulating the imbalance of gut microbiota, inhibiting Toll-Imd pathways to attenuate the excessive immune response, and inhibiting JAK-STAT pathway to alleviate inflammation. In addition, formononetin, kaempferol and ergosterol are the major compounds of GFXD to alleviate intestinal injury caused by CPT-11 (Fig. 11).

Dunhuang Gancao Fuling Xingren decoction (GFXD) originated from the *Dunhuangyishu P.3596*, is traditionally known for its immunomodulatory and anti-inflammatory properties [16–18]. In this study, supplementation of GFXD alleviated the body injury in CIM flies. Consistently, the similar phenotype was observed in mice that administration of GFXD notably attenuated CPT-11-induced intestinal mucosal injury and inhibited the expression of inflammatory factors. CIM is the most common chemotherapeutic drug-induced gastrointestinal toxicity, and patients present with symptoms of diarrhea, hematochezia, and appetitive dysfunction [36]. Furthermore, GFXD significantly increased the length of intestine, and alleviated acid–base imbalance in CPT-11-induced flies. Consistently, GFXD could increase gut length and restore crypt disappearance and goblet cell depletion in CPT-11 mice. CPT-11 administration led to a significant decrease of the intestinal stem and intestinal epithelial cells. TJ proteins ZO-1 and occludin play a crucial role in mucosal repair, and their expression levels are closely linked to intestinal epithelial barrier permeability [37, 38]. This study demonstrated that GFXD treatment effectively reversed the downregulation of ZO-1 and occludin in colon tissue, suggesting a potential mechanism by which GFXD may protect the intestinal barrier in mice. In conclusion, the present study comprehensively demonstrates the protective efficacy of GFXD against CPT-11-induced intestinal injury in both Drosophila and mouse models, elucidating its multi-faceted mechanisms of action.

Transcriptomic analysis revealed that therapeutic effects of GFXD were primarily associated with several key pathways, including the defense response to bacteria, Toll-Imd, and JAK-STAT signaling pathways. The imbalance of the intestinal microbiota is considered to play a key role when exploring the specific mechanisms leading to CIM [39]. Here, CPT-11 could change the structure and composition of the microbial community in the intestine, result in a reduction in the number of beneficial bacteria that were originally in a balanced state, while harmful bacteria or opportunistic pathogens take the opportunity to multiply in large numbers. Numerous studies have shown that CIM is associated with altered gut microbiota immune reaction and inflammation [40, 41]. Our findings from the FMT and ABX treatment experiments suggest that the gut microbiota plays a critical role in intestinal repair. Gut microbiome analysis identified six bacterial genera significantly associated with CPT-11-induced damage and GFXD intervention: *Bacteroides*, *Enterococcus, Helicobacter, Enterobacter, Lactobacillus* and *Prevotella*. As a currently recognized probiotic genus, *Lactobacilli* is able to improve intestinal barrier function, regulate the immune, and exert anticancer activity [42]. *Bacteroides* is different from *Lactobacillus* genus. Although there are probiotics like *Bacteroides acidifaciens* in *Bacteroides*, yet pathogenic bacteria species, such as *enterotoxigenic B. fragilis,* which may stimulate colitis or even systemic inflammation [43]. *Enterococcus*, *Helicobacter* and *Enterobacter* are usually regarded as pathogenic bacteria or conditioned pathogens. For example, increased *Bacteroides* levels induced colitis in mice. *Helicobacter* as a pathogen of intestinal diseases would cause inflammation of the gastrointestinal system and led to peptic ulcers [44, 45]. *Helicobacter pylori* infection caused peptic ulcer disease or even gastric cancers [46, 47]. In the present study, the relative abundances of *Lactobacillus* and *Prevotella* sharply decreased, while others increased in the CPT-11 group, which was rescued after GFXD treatment [48], which was consistent with previous findings [9, 49].

The Toll-Imd signaling pathways are crucial for recognizing pathogen-associated molecular patterns and are activated by the gut microbiota [50, 51]. The Imd pathway plays a key role in immune defense of *Drosophila*, and has a similar function in the intestinal immunity of mammals [52]. In this study, CPT-11 activated the Imd pathway and regulated the expression of immune-related genes, enhanced the activation of immune cells and the release of inflammatory mediators, and promoted the development of CIM in coordination with the Toll pathway. In addition, the JAK/STAT signaling pathway is primarily regulated by cytokines, plays a critical role in mediating innate immune activation while simultaneously modulating inflammatory and immune responses [53, 54]. When intestinal inflammation occurs, cytokines bind to their corresponding receptors, activating JAK kinase, which then phosphorylate STAT proteins and transfer them into the nucleus, regulating the expression of a series of genes related to inflammatory responses, cell proliferation, and survival. CPT-11 affects the secretion and signal transduction of cytokines such as IL-6 [55], which may activate the JAK1/STAT3 pathway to continuously activate the inflammatory cells, thereby aggravating the degree of intestinal mucositis [56]. To conclude, the mechanism by which GFXD treats CPT-11-induced CIM is a complex pathological process involving modulating of gut microbiota, Toll-Imd pathways, JAK/STAT pathway. In addition, transcriptome analysis demonstrated that GFXD also modulated metabolic pathways, such as tyrosine metabolism, sphingolipid metabolism, and glutathione metabolism, and molecular functions (iron ion binding) in CPT-11-induced flies, which need to be explored in the future.

Through LC–MS screening and experimental validation, we identified formononetin, kaempferol, and ergosterol could exhibit the most pronounced efficacy in alleviating CIM. Previous studies indicate that formononetin is a type of phytoestrogen which mitigates inflammatory effects by JAK2/STAT3 pathway inhibition and reduce oxidative stress [57, 58]. Kaempferol is an anti-inflammatory and antioxidant flavonoid which could restore gut microbiota and inhibit the LPS-TLR4-NF-κB axis, and demonstrated anticancer properties [59, 60]. Ergosterol is a characteristic sterol of fungi with various bioactive functions which could reduce the inflammation-related intestinal microbial genera, while reduce lipid levels and suppress pro-inflammatory responses, attenuates oxidative damage, modulation of apoptosis and tight junction in the small intestine [61–63]. These findings provide mechanistic insights to optimize GFXD's clinical translation for chemotherapy-induced intestinal mucositis management.

In conclusion, this study validated the protective effect of GFXD against CPT-11-induced CIM using both fly and mouse models. The pathogenesis of GFXD against CIM is mediated mainly by inhibiting the Toll-Imd and JAK-STAT pathways, alongside the modulation of gut microbiota dysbiosis. Meanwhile, formononetin, kaempferol and ergosterol were identified as the major compounds of GFXD against CIM. Taken together, our findings demonstrate that GFXD is a novel multi-target therapeutic agent for CIM management, thereby establishing a translational bridge between Dunhuang medical wisdom and modern evidence-based precision medicine.

## Data Availability

No datasets were generated or analysed during the current study.

## Abbreviations

7-AAD: 7-Amino actinomycin D
AB-PAS: Alcian Blue-periodic Acid Schiff Staning
ANOVA: Analysis of variance
Ara-C: Cytarabine
CCR: Copper cell region
CIM: Chemotherapy-induced intestinal mucositis
COPD: Chronic obstructive pulmonary disease
CPT-11: Irinotecan
DAI: Disease activity index
DAPI: 4′ 6-Diamidino-2-phenylindole
DCM: Dilated cardiomyopathy
DEGs: Differentially expressed genes
ELISA: Enzymelinked immunosorbent assay
FMT: Fecal microbiota transplantation experiment
GFP: Green fluorescent protein
GFXD: Dunhuang Gancao Fuling Xingren decoction
GO: Gene ontology
H&E: Hematoxylin and eosin staining
IL-10: Interleukin-10
IL-1β: Interleukin-1 beta
IL-6: Interleukin-6
JAK: Janus Kinase
KEGG: Kyoto encyclopedia of genes and genomes
LC–MS: Liquid chromatograph mass spectrometer
PBS: Phosphate-buffered Saline
PBST: Phosphate-buffered saline with tween
PCA: Principal component analysis
PCoA: Principal coordinates analysis
qRT-PCR: Quantitative Real-time PCR
Rp49: Ribosomal protein
SPF: Specific pathogen-free
STAT: Signal transducer and activator of transcription
TCM: Traditional Chinese medicine
TNF-α: Tumor necrosis factor-alpha
